# Association between serum and dietary antioxidant micronutrients and advanced liver fibrosis in non-alcoholic fatty liver disease: an observational study

**DOI:** 10.7717/peerj.9838

**Published:** 2020-09-17

**Authors:** Juliana Moraes Coelho, Katia Cansanção, Renata de Mello Perez, Nathalie Carvalho Leite, Patrícia Padilha, Andrea Ramalho, Wilza Peres

**Affiliations:** 1 The Capriglione Luiz State Institute of Diabetes and Endocrinology, Rio de Janeiro, Rio de Janeiro, Brazil; 2 Universidade Federal do Rio de Janeiro, Rio de Janeiro, Brazil; 3 Universidade Federal do Rio de Janeiro and D’Or Institute for Research and Education, Rio de Janeiro, Brazil; 4 Department of Internal Medicine, Universidade Federal do Rio de Janeiro, Rio de Janeiro, Brazil; 5 Department of Social and Applied Nutrition and Center for Research on Micronutrients (NPqM), Universidade Federal do Rio de Janeiro, Rio de Janeiro, Brazil

**Keywords:** NAFLD, Liver fibrosis, Elastography, Antioxidants, Micronutrients, Retinol, Vitamin C, Vitamin E, Selenium, Zinc

## Abstract

**Background:**

Despite clinical trials with antioxidant supplementation, few studies have been conducted to evaluate the nutritional status of antioxidant vitamins and minerals, and none have reported on the status of these serum antioxidants associated with the dietary intake of antioxidants by non-alcoholic fatty liver disease (NAFLD) patients.

**Objective:**

To evaluate association between serum and dietetics antioxidants with liver fibrosis in patients with NAFLD.

**Methods:**

Across-section analysis with out with 72 patients diagnosed with NAFLD. Hepatic fibrosis was measured by FibroScan^®^, and liver stiffness ≥7.9 kPa was considered to indicate advanced fibrosis. Retinol, alpha-tocopherol, ascorbic acid, beta-carotene, serum zinc, and selenium were evaluated, as was the dietary intake of these micronutrients in the previous 24 h (using 24-h dietary recall). The Mann–Whitney test was used to compare the fibrosis groups and, a linear regression analysis was performed to determine associated risk factors between age, sex, BMI, hepatic fibrosis, and serum antioxidants.

**Results:**

A high proportion of inadequate serum retinol (20.8%), vitamin C (27%), and selenium (73.6%) was observed in the patients with NAFLD, in addition to a significant inadequacy of vitamin A (98.3%) and vitamin E (100%) intake. Patients with advanced liver fibrosis had reduced levels of serum retinol (*P* = 0.002), with liver fibrosis being the independent risk factor associated with serum retinol lower.

**Conclusion:**

Hepatic fibrosis was associated with a reduction in serum retinol and was reduced in advanced fibrosis. NAFLD patients showed an important serum deficiency and insufficient dietary intake of the evaluated micronutrients.

## Introduction

Non-alcoholic fatty liver disease (NAFLD) has emerged in recent years as a leading cause of chronic liver disease (CLD) in Western countries (Younossi et al., 2016). The global prevalence in the general population is approximately 25%, reaching as high as 80% in obese individuals (Williams et al., 2011; Younossi et al., 2016). NAFLD encompasses a spectrum ranging from simple steatosis to non-alcoholic steatohepatitis (NASH), with or without fibrosis, which can progress to cirrhosis and hepatocellular carcinoma (Chalasani et al., 2018).

Because it is a complex disease, several factors are related to its progression, although oxidative stress seems to play a key role, contributing to lipid peroxidation, inflammation, and activation of hepatic stellate cells (Rolo, Teodoro & Palmeira, 2012; Gambino, Musso & Cassader, 2016). Under normal conditions, aerobic hepatic metabolism involves the production of pro-oxidants, such as reactive oxygen and nitrogen species, which are compensated by a similar rate of antioxidants (Ferramosca, Di Giacomo & Zara, 2017), but in NAFLD there is an imbalance between these components (Videla et al., 2004). Increased markers of lipid peroxidation (Loguercio et al., 2004), increased proinflammatory cytokines (Loguercio et al., 2004; Jamali et al., 2016) and reduced total plasma antioxidant capacity have been shown in NAFLD (Tilg & Moschen, 2008).

Faced with this scenario, many clinical trials have evaluated the efficacy of supplementation with antioxidant vitamins, especially vitamin E, which were found to promote improvements in liver fibrosis in a study conducted by Sanyal et al. (2004), and promoted a significant improvement in the liver fibrosis score of patients with NASH when associated with vitamin C (Harrison et al., 2003).

However, despite clinical trials with antioxidant supplementation, few studies have been conducted to evaluate the nutritional status of antioxidant vitamins and minerals, and none have reported on the status of these serum antioxidants associated with the dietary intake of antioxidants by NAFLD patients (Koruk et al., 2004; Yesilova et al., 2005; Cankurtaran et al., 2006).

In view of the importance of antioxidant action in the progression of NAFLD and the scarcity of studies relating antioxidants to hepatic fibrosis, which is an independent factor for prediction of death in NAFLD (Ekstedt et al., 2015; Angulo et al., 2015; Golabi et al., 2018), the objective of this study was to evaluate association between serum and dietary antioxidant micronutrients with advanced liver fibrosis in patients with NAFLD.

## Materials and Methods

### Study design

This was a cross-sectional observational study with the prospective inclusion of patients attending the outpatient the hepatology clinic of Clementino Fraga Filho University Hospital (HUCFF), part of the Federal University of Rio de Janeiro, between August 2016 and December 2017. The protocol of this study was approved by the Research Ethics Committee of HUCFF, under registration (Certificate of Presentation for Ethical Assessment–CAAE: 18379713.4.0000.5257), according to the terms of National Health Council (Conselho Nacional de Saúde) resolution 196. All participants were included by signing informed consent and a convenience sample was obtained for this study.

### Study population

Patients older than 19 years of both sexes who had been diagnosed with NAFLD by ultrasonography were included (Chalasani et al., 2018). All these patients then underwent a FibroScan^®^ transient hepatic elastography (THE) to quantify fibrosis. Exclusion criteria were hepatitis B and C infection, autoimmune liver disease, alcohol intake >30 g/day for men and >20 g/day for women, human immunodeficiency virus (HIV) infection, other infections, renal failure, liver transplant, cancer, pregnancy or breastfeeding, use of vitamin or mineral supplements in the previous 6 months, and use of tamoxifen, methotrexate, glucocorticoids, amiodarone, synthetic estrogens, diltiazem, or acetylsalicylic acid.

### Measurement of liver stiffness using transient elastography

All the patients underwent a THE scan using the FibroScan^®^–EchoSens (Paris, France), which was conducted by a single experienced examiner. The examinations were performed with the patients in dorsal decubitus, with their right arm in maximum abduction. The site for the measurement of liver stiffness was the sixth or seventh intercostal space, in the right mid-axillary line. Only the exams with 10 valid measures, a success rate of over 60%, and an interquartile range of less than 30% were considered. Probe XL was used when probe M was unable to measure liver stiffness (Eddowes et al., 2019).

The median value was considered representative of the measure of liver elasticity and this was the variable recorded in relation to elastography. The results were expressed in kilopascals (kPa), ranging from 1.5 to 75 kPa, and median hepatic fibrosis was categorized into two groups: liver elastography <7.9 kPa without advanced fibrosis and liver elastography ≥7.9 kPa with advanced fibrosis. This cutoff point was based on a study that proposed 7.9 kPa as the optimum cutoff point for the diagnosis of advanced fibrosis in patients with NAFLD (Wong et al., 2010).

### Biochemical assessments

Samples of five ml blood were collected after fasting for 12 h to evaluate serum antioxidants. Quantification of serum vitamins was performed using high-performance liquid chromatography with ultraviolet detector. The cutoff points used to classify vitamin inadequacy were: retinol <1.05 μmol/L, vitamin C <4.6 mg/L, vitamin E <5 mg/L, and beta-carotene <40 ng/mL (Viulleumier et al., 1983; Lee, Roberts & Labbe, 1997; International Vitamin A Consultative Group (IVACG), 2003). Serum zinc levels were determined by the colorimetric method (ZN23410; Randox^®^, Crumlin, UK) and the cutoff point for deficiency was zinc <70 μg/dL (Sauberlich, Dowdy & Skala, 1973). Selenium was determined by atomic absorption spectrophotometry (Analyst 600; Perkin Elmer^®^, Waltham, MA, USA) and the cutoff point was selenium <91.51 μg/L (Burk, 2002).

All analyses were performed at the Richet clinical analysis laboratory in Rio de Janeiro, Brazil. Total cholesterol, LDL, HDL, triglycerides (TG), glucose, glycated hemoglobin, gamma glutamyl transferase (GGT), aspartate aminotransferase (AST), alanine aminotransferase (ALT) and alkaline phosphatase (ALP) were obtained from routine medical follow-up.

### Evaluation of dietary intake of micronutrients

Data on micronutrient intake were obtained through the 24-h dietary recall (24HR), applied at three different times, one on an atypical day, using the five multiple pass method according (Conway et al., 2003), being carried out by interviewers previously trained and in a room designed for this purpose (Willett, 1998).

Dietary micronutrient intake was estimated for three nonconsecutive days using 24HR. A specific data entry program was used, consisting of approximately 1,500 items (food and beverages) selected from 5.686 records based on food and beverage acquisition data from the Household Budget Survey conducted by the Instituto Brasileiro de Geografia e Estatística, IBGE, in 2008–2009. The survey also includes codes for recording the way the food was prepared (14 cooking methods) and the unit of measurement used to report the amount consumed (106 home measures).

To estimate the intake of micronutrients, the tables of nutritional composition and home measures, compiled specifically to analyze the foods and preparations listed in the Household Budget Survey 2008–2009 were used (Instituto Brasileiro de Geografia e Estatística (IBGE), 2011a, 2011b). The results of the nutritional composition for the three 24HR were shrunk to correct for intra-individual variability using the Multiple Source Method (https://msm.dife.de) (Harttig, Haubrock & Knüppel, 2011). In order to evaluate the adequacy of dietary micronutrient intake, the dietary reference intakes were used, taking into account the estimated average requirement (Institute of Medicine (IOM), 2000; Institute of Medicine (IOM), 2001).

### Anthropometric assessment

Body weight was measured using a FILIZOLA^®^ mechanical scale with a maximum capacity of 150 kg and a precision of 100 g. Height was measured using a stadiometer, with the individual standing barefoot with their heels together, back straight, and arms extended at the side of their body. Body mass index (BMI) was calculated by the formula: weight (kg)/height/m^2^. Waist circumference (WC) was measured at the midpoint between the last rib and the iliac crest and was classified according to the World Health Organization (WHO) (2011).

### Statistical analysis

Statistical analysis was performed using the Statistical Package for Social Sciences (SPSS), version 21.0. The normality of the sample distribution was assessed using the Shapiro–Wilks test. The descriptive analysis presented the observed data in the form of tables, expressed by measures of central tendency and dispersion (median and interquartile range) for numerical data and frequencies (*n*) and percentages (%) for categorical data. The Mann–Whitney test was used to compare the fibrosis groups with the anthropometric, biochemical, serum antioxidant, and dietary variables. In addition, simple and multivariate linear regression analyses, including the variables age, sex, BMI, hepatic fibrosis, and serum antioxidants, these variables were log transformed before analysis.

## Results

**Figure 1:**
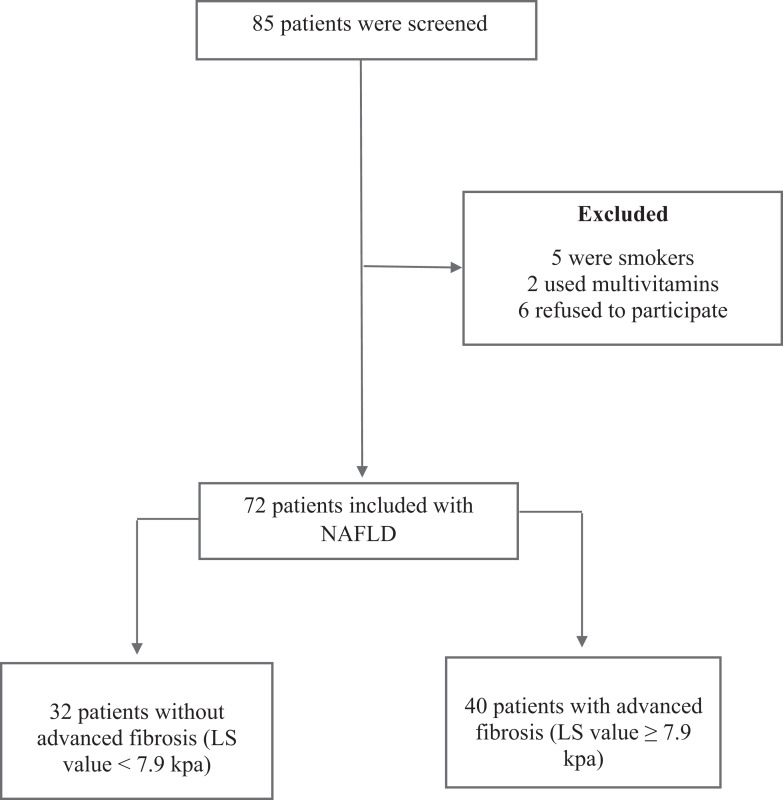
Flowchart. Notes: NAFLD, Non-alcoholic Fatty Liver Disease; LS, liver stiffness; kPa, Kilopascals.

A total of 85 patients were screened, however, 5 were smokers, 2 used multivitaminics and 6 refused to participate. Thus, 72 patients included, 55.5% or (*N* = 40) of whom had advanced fibrosis (liver stiffness ≥ 7.9 kPa) and, 44.5% or (*N* = 32) without advanced fibrosis (liver stiffness <7.9 kPa) (Fig. 1).

The median age was 59 (28–81) years and a predominance of females, representing 77.8% or (*N* = 56) of the total of individuals evaluated. A high proportion of NAFLD patients showed inadequate serum concentration of micronutrients with antioxidant function, especially selenium (73.6% or *N* = 53), vitamin C (27% or *N* = 19), and vitamin A (20.8% or *N* = 15) (Table 1).

**Table 1:** Proportion of individuals with NAFLD who had inadequate micronutrients status inadequacy of serum antioxidants.

Antioxidants	Cutoff points	Median	IQR	Inadequacy (%) (*N*)
Retinol	<1.05 µmol/L	1.36	1.11–1.81	20.83 (15)
Vitamin C	<4.6 mg/L	5.72	3.24–8.44	27.00 (19)
Vitamin E	<5 mg/L	10.15	8.05–12.72	1.38 (1)
Betacarotene	<40 ng/mL	178.05	104.60–278.90	1.44 (1)
Selenium	<91.51 µg/dL	81.50	71.25–92.00	73.61 (53)
Zinc	<70 µg/dL	87.25	78.72–96.65	18.61 (13)

**Note:**

IQR, interquartile range.

The patients with advanced fibrosis had a significantly higher BMI than those without advanced fibrosis (*P* = 0.023). Retinol was the only serum antioxidant that was significantly lower in the group with advanced fibrosis than the group without advanced fibrosis (*P* = 0.002). There was no statistically significant difference in relation to micronutrient intake in the different fibrosis groups (Table 2).

**Table 2:** Comparison of anthropometric, biochemical and serum and dietary antioxidant micronutrient variables with degree of liver stiffness.

Variables	Patients without advanced fibrosis (^#^LS value <7.9 kPa) *N* = 32		Patients with advanced fibrosis (^#^LS value ≥7.9 kPa) *N* = 40		
	Median	IQR	Median	IQR	P*
Age (years)	59.00	56.00–66.75	60.50	55.00–67.00	0.738
BMI (kg/m^2^)	29.77	26.93–35.10	34.28	29.87–38.06	0.023*
WC (cm)	103.50	94.37–108.75	109.50	98.25–117.75	0.051
Serum Concentration					
Glucose (mg/dL)	98.00	89.50–118.00	111.50	95.25–133.75	0.065
Hb A1C (%)	6.10	5.90–6.50	6.30	5.80–7.90	0.340
ALT (U/L)	42.50	32.25–48.75	50.50	36.25–81.00	0.034*
AST (U/L)	27.50	21.25–34.75	38.00	24.25–55.75	0.002*
AST/ALT	0.57	0.52–0.71	0.65	0.52–0.89	0.196
GGT (U/L)	40.00	28.25–52.25	98.50	52.50–161.00	<0.001*
ALP (U/L)	103.00	79.00–122.50	109.00	92.00–127.00	0.398
Total cholesterol (mg/dL)	171.50	156.50–216.25	176.00	147.25–191.00	0.236
LDL-c (mg/dL)	92.00	76.75–131.00	85.00	66.25–108.50	0.196
HDL-c (mg/dL)	48.00	43.00–55.00	45.00	38.00–57.50	0.444
Triglycerides (mg/dL)	119.50	80.00–184.25	108.50	82.50–164.50	0.816
Retinol (µmol/L)	1.57	1.24–1.84	1.16	0.69–1.66	0.002*
Vitamin C (mg/L)	5.16	3.54–7.21	7.03	2.78–9.35	0.219
Vitamin E (mg/L)	10.20	8.00–13.40	10.00	8.22–12.00	0.533
βetacarotene (ng/mL)	196.10	101.62–306.70	170.00	106.60–271.10	0.713
Zinc (µg/dL)	89.75	79.37–99.32	85.30	76.80–95.02	0.324
Selenium (µg/L)	82.00	76.00–93.75	77.50	66.25–91.00	0.103
Dietary Intake					
Vitamin A (µg)	222.28	156.28–290.92	201.56	148.56–246.90	0.167
Vitamin C (mg)	68.93	47.19–88.28	80.90	44.23–109.73	0.434
Vitamin E (mg)	3.71	2.46–5.09	3.36	2.75–4.14	0.287
β-carotene (mg)	2033.46	1114.76–2540.18	1405.94	652.03–2449.12	0.181
Zinc (mg)	7.86	6.63–9.84	7.72	6.41–8.93	0.475
Selenium (µg)	56.60	48.10–67.87	53.80	47.97–63.82	0.458

**Notes:**

# LS, liver stiffness; BMI, body mass index; WC, waist circumference; ALT, alanine transaminase; AST, aspartate transaminase; GGT, gamma-glutamyl transferase; ALP, alkaline phosphatase; HbA1c, glycated hemoglobin; LDL-c, low-density lipoprotein-cholesterol; HDL-c, high-density lipoprotein-cholesterol.

* *P* < 0.05 is significant.

Values are medians (IQRs, interquartile ranges). Comparison were done by Mann–Whitney *U* test.

**Figure 2:**
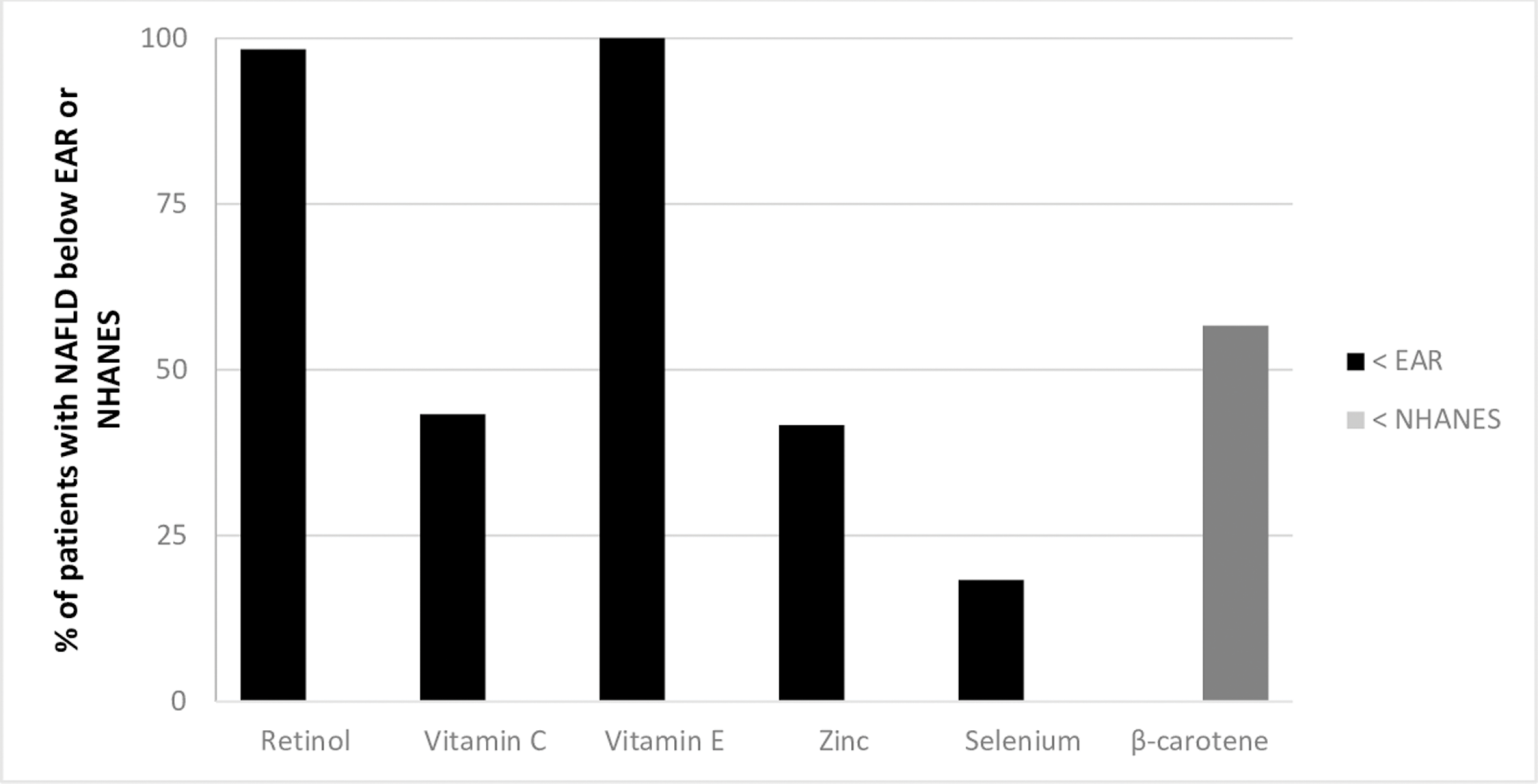
Prevalence of inadequacy of antioxidant micronutrients in patients with NAFLD. Percentage inadequacy of consumption of micronutrients in patients with NAFLD, based on estimated average requirement (EAR) and mean values (habitual intake) of all the individuals, regardless of gender or age group (NHANES III, 1988–1994); Institute of Medicine.

In addition, the dietary intake of micronutrients was insufficient in most of the patients with NAFLD, with levels below the EAR amongst 98.3% or (*N* = 71) of the patients for vitamin A, 43.3% or (*N* = 26) for vitamin C, 100% or (*N* = 72) for vitamin E, 18.05% or (*N* = 13) for selenium, 41.6% or (*N* = 30) for zinc, and 56.6% or (*N* = 40) for beta-carotene, according to NHANES III (Fig. 2).

In the Table 3 shows the results of the simple linear regression between the serum micronutrients and age, gender, BMI (kg/m^2^) and liver stiffness (kPa). Using a multiple linear regression model it was observed that liver fibrosis was inversely associated with serum retinol concentration. The other serum micronutrients did not present a significant contribution in the model (Table 3).

**Table 3:** Factors associated with low serum micronutrients with antioxidant function.

Variables	Univariate	Multivariate			
	*P*	β		CI 95%	*P*
Retinol					
Age (years)^#^	0.195	0.204		[−0.001 to 0.025]	0.075
Gender^#^	0.355	−0.115		[−0.457 to 0.146]	0.307
BMI (kg/m^2^)^#^	0.259	−0.047		[−0.027 to 0.018]	0.689
Liver stiffness (kPa)^#^	0.005	−0.346		[−0.046 to −0.009]	0.004*
Vitamin C					
Age (years)^#^	0.187	0.148		[−0.036 to 0.148]	0.227
Gender^#^	0.520	−0.084		[−2.827 to 1.361]	0.487
BMI (kg/m^2^)^#^	0.649	−0.84		[−0.205 to 0.102]	0.506
Liver stiffness (kPa)^#^	0.506	0.082		[−0.087 to 0.172]	0.515
Vitamin E					
Age (years)^#^	0.274	−0.111		[−0.109 to 0.039]	0.349
Gender^#^	0.049	−0.234		[−3.398 to −0.009]	0.049
BMI (kg/m^2^)^#^	0.595	−0.044		[−0.147 to 0.102]	0.718
Liver stiffness (kPa)^#^	0.223	−0.117		[−0.155 to 0.055]	0.343
βetacarotene					
Age (years)^#^	0.172		−0.172	[−8.699 to 1.521]	0.165
Gender^#^	0.681		−0.033	[−133.146 to 101.371]	0.788
BMI (kg/m^2^)^#^	0.021		−0.151	[−13.326 to 3.370]	0.238
Liver stiffness (kPa)^#^	0.908		0.039	[−5.977 to 8.132]	0.761
Zinc					
Age (years)^#^	0.997		0.005	[−0.560 to 0.582]	0.970
Gender^#^	0.090		−0.207	[−24.389 to 1.737]	0.088
BMI (kg/m^2^)^#^	0.549		−0.086	[−1.292 to 0.628]	0.492
Liver stiffness (kPa)^#^	0.526		−0.008	[−0.833 to 0.781]	0.950
Selenium					
Age (years)^#^	0.165		0.149	[−0.161 to 0.705]	0.214
Gender^#^	0.150		−0.174	[−17.224 to 2.590]	0.145
BMI (kg/m^2^)^#^	0.706		−0.003	[−0.737 to 0.719]	0.980
Liver stiffness (kPa)^#^	0.202		0.133	[−0.283 to 0.941]	0.287

**Notes:**

# Variables were log transformed before analysis. BMI, body mass index.

* *P* < 0.05 is significant.

Multivariate analysis was performed using a linear regression model.

## Discussion

In the present study, retinol deficiency was considered an independent risk factor associated with liver fibrosis in a reversible stage of liver disease (NAFLD). In addition, a high frequency of serum retinol, selenium and vitamin C deficiency and a high prevalence of inadequate intake of vitamin A, vitamin E, vitamin C, selenium, zinc and betacarotene were found. This is the first study that endeavors to investigate the simultaneously serum levels of retinol, vitamin E, vitamin C, betacarotene, zinc and selenium according to biochemical, and dietetic indicators in NAFLD, in individuals with and without advanced liver fibrosis.

Vitamin A deficiency (VAD) was 20.30% in total sample and 31.9% in advanced fibrosis stage. It is noteworthy that patients without advanced fibrosis had no VAD. In previous studies, the prevalence of VAD were 27.6% in chronic hepatitis (Peres et al., 2011), 60% in liver pre transplant patients and 42.5% in patients with cirrhosis Child A (De Paula, Ramalho & Braulio, 2010). These data reveal that VAD in NAFLD patients with advanced stage of fibrosis was higher than VAD found in patients with early stage of CLD, that is, chronic hepatitis, but the frequency of VAD was lower than that found in more advanced stages of CLD.

Additionaly, dietary vitamin A intake is notably insuficient in almost 100% of the sample. Poor vitamin A intake leads to depletion of retinol stocks and may contribute to the progression of liver disease, as oxidative stress is an important pathophysiological mechanism in NAFLD patients and retinol is recognized to have important antioxidant action in liver disease (Fierbinteanu-Braticevici et al., 2009). In fact, Lotfi et al. (2019) demonstrated reduced risk of NAFLD in individuals with higher vitamin A intake compared to those with lower intake.

In our study, a significant reduction in serum retinol was observed in patients with advanced hepatic fibrosis and retinol was the only independent factor associated with advanced liver fibrosis. This finding is in line with previous findings (Newsome et al., 2000; De Paula, Ramalho & Braulio, 2010; Peres et al., 2011), where VAD was associated with the severity of CLD and hepatocellular carcinoma in cirrhotic patients, corroborating that the progression of CLD is associated with reduced serum retinol levels. Considering that about 80% of the retinol reserve is stored in hepatic stellate cells (HSC), and oxidative stress activates these cells by modifying them in collagen-producing myofibroblasts, the perpetuation of liver injury culminates in loss of storage capacity of retinol (Saeed et al., 2017).

Recently, using data from the Nutrition and Health Examination Surveys has showed that the higher the quartile of seric retinol the lower the risk for NAFLD (Mazidi, Huybrechts & Kegne, 2019). However, a study case control found no difference in retinol concentrations of NAFLD patients compared to healthy individuals, possibly due to lack of stratification by the NAFLD spectrum (Tayyen, Al-Dayyat & Rayyan, 2019).

We have observed that all individuals in the sample had lower than recommended vitamin E intake, while they still had normal serum levels in most patients. Although the perpetuation of insufficient intake could lead to deficiency of this important lipid phase antioxidant, contributing to the progression of NAFLD. An earlier study by Cortez-Pinto et al. (2006) corroborate the high proportion of inadequate vitamin E intake (75.6%) in Portuguese with NAFLD, however, the study did not evaluate serum micronutrient concentrations.

As for serum ascorbic acid, we observed a high prevalence of deficiency compared to previous studies with a western population (Hampl, Taylor & Johnston, 2004; Lykkesfeldt & Poulsen, 2010). These findings can be explained by the low consumption of the vitamin C in the population of the present study, as well as the possible use of ascorbic acid in the vitamin E regeneration pathway, as tocopheryl radical may react with ascorbate in the presence of selenium or sulfur (Furuse, 1987). Previous study carried out in Canada found low vitamin C intake in patient with NAFLD, ranging from 25% to 35%, lower than found in the present study (Da Silva et al., 2014). As far as we know, there are no published data regarding the proportion of inadequate serum vitamin C in adults with NAFLD.

Additionally, a high proportion of NAFLD patients showed serum selenium deficiency, even with insufficient intake in only 18.05% of the total sample. This discrepancy may be related to the increased demand for serum selenium for its antioxidant and antiinflammatory effects, maintenance of redox homeostasis and for regeneration of vitamin E (Rayman, 2012; Polyzos et al., 2019), suggesting that the requirement for selenium for patients with NAFLD is higher than that recommended for healthy individuals.

In contrast, despite the high proportion of inadequate intake, zinc presented low serum deficiency. Ninety percent of zinc content is concentrated in bones, voluntary muscles, liver and skin, suggesting that normal serum values are being maintained at the expense of zinc reserve. Zinc is involved in retinol binding protein synthesis, where it promotes the binding of transcription factors to deoxyribonucleic acid (DNA) for synthesis of this protein (Vallee & Falchuk, 1993), and low intake may be contributing to VAD in NAFLD patients, since reduced retinol levels could be related to zinc deficiency (Smith, 1980).

We have noted that more than half of the patients presented low betacarotene consumption. Despite the potential of carotenoids as potent antioxidants and anti inflammatories, no studies were found on the adequacy of betacarotene consumption in NAFLD (Eggersdorfer & Wyss, 2018), what hampers the discussion. However, low betacarotene intake may be impacting serum retinol levels as absorbed betacarotene may be converted to retinol in the enterocyte by the betacarotene 15-15′ monooxygenase enzyme (Lindqvist & Andersson, 2002).

We also observed levels significant high in of ALT, AST, and GGT in individuals with liver advanced fibrosis. In fact, previous studies have shown significantly elevated levels of these liver enzymes in this advanced liver fibrosis (Cansanção et al., 2018; Caussy et al., 2019), reinforcing its clinical utility. However, it should be noted that normal levels of these enzymes do not exclude the diagnosis of advanced liver fibrosis.

Possible limitations of the present study may be the non-use of liver biopsy for histological staging of NAFLD, however, even though it is considered the gold standard, it is an invasive method and may have limitations related to the evaluator and sample. Being THE considered an increasingly used method for monitoring liver fibrosis in NAFLD.

## Conclusion

The present study, the serum retinol was associated with advanced liver fibrosis and, a high proportion of NAFLD patients showed serum deficiency of retinol, vitamin C and selenium. In addition, we highlight the important inadequacy in the consumption of the evaluated micronutrients. Those finding suggests that retinol is an important marker of liver disease progression even in the possible stages of liver disease reversal, and that these population should be the target of nutritional intervention strategies, paying special attention to food sources of retinol and vitamin E, since the all patients evaluated presented consumption below the recommended.

## Supplemental Information

10.7717/peerj.9838/supp-1 Supplemental Information 1 Dataset.

## Abbreviations

NAFLD: Non-alcoholic Fatty Liver Disease
CLD: Chronic Liver Disease
NASH: Non-Alcoholic Steatohepatitis
HUCFF: Clementino Fraga Filho University Hospital
THE: Transient Hepatic Elastography
kPa: Kilopascals
HIV: Human Immunodeficiency Virus
24HR: 24-Hour Dietary Recall
IBGE: Instituto Brasileiro de Geografia e Estatística
BMI: Body Mass Index
WC: Waist Circumference
VAD: Vitamin A Deficiency
HSC: Hepatic Stellate Cells
DNA: Deoxyribonucleic Acid

## Additional Information and Declarations

### Data Availability

The following information was supplied regarding data availability: Raw data is available as a Supplemental File.
